# Stochastic models of routing strategies under the class-based storage policy in fishbone layout warehouses

**DOI:** 10.1038/s41598-022-17240-w

**Published:** 2022-07-27

**Authors:** Li Zhou, Junhui Zhao, Huwei Liu, Fan Wang, Jianglong Yang, Senhao Wang

**Affiliations:** 1 School of Information, Beijing Wuzi University, Beijing, 101149 China; 2 School of Management and Engineering, Capital University of Economics and Business, Beijing, 100070 China

**Keywords:** Applied mathematics, Computational science, Computer science

## Abstract

In order to improve the picking efficiency of warehouses, shorten the time cost and promote the development of the logistics industry, this study analyzes the routing strategies in fishbone layout warehouses under the class-based storage strategy. The fishbone layout was divided into three storage areas for class A, class B, and class C items according to the proportion using the straight line, to meet the classification requirements of items. Under the class-based storage strategy, to evaluate the performance of the return routing strategy and the S-shape routing strategy, the stochastic models of the expected walking distance of the two routing strategies in the fishbone layout warehouse are established by calculating the sum of the expected walking distances in diagonal cross-aisles and picking aisles. Finally, the stochastic models of the two routing strategies are simulated and verified, and the impacts of the two routing strategies on walking distances are analyzed by comparing the expected distances under different ordering frequencies and space allocation strategies. The numerical results show that the return routing strategy has an advantage over the S-shape routing strategy when determining the relevant parameters of the fishbone layout and picking orders. Meanwhile, it also provides a theoretical basis for research on stochastic models of routing strategies in fishbone layout warehouses under the class-based storage strategy.

## Introduction

Warehousing is essential to logistics and supply chains^1^. Similarly, picking is an indispensable part of warehousing activities and is a major factor affecting the efficiency of warehouses and distribution centers^2^. In operation cost, the proportion of picking activities in total warehouse costs can reach 50–75%^3–8^, and in time cost, the walking or traveling time also accounts for more than 50% of the total picking operation time^5,9,10^. In addition, some studies have found that factors such as warehouse layout, storage strategy, and routing strategy have an impact on the picking efficiency in warehouses^11^. Of them, the warehouse layout significantly affects the total picking travel distance, and the difference may be greater than 60%^12^. Therefore, a good storage strategy or warehouse layout can reduce the traveling distances between in and out of the warehouse, shorten the operation time, and make full use of the storage space.

**Figure 1:**
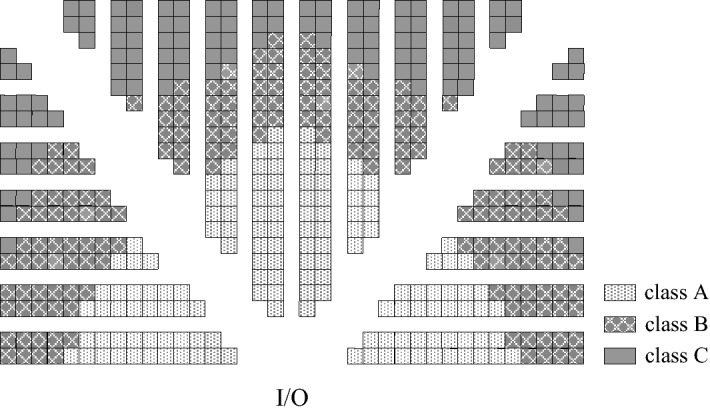
Fishbone layout warehouse under the class-based storage strategy (created with Microsoft Visio Professional 2019, 16.0.14827.20216, https://www.microsoft.com/en-gb/microsoft-365/visio/flowchart-software).

At present, most researchers mainly discuss the selection and optimization of the picking strategy, storage strategy and routing strategy under the traditional layout. In actual warehouse operations, the shelf layout is also the traditional layout. To solve the storage problem, the non-traditional warehouse layout has emerged as a new idea. Gue and Meller^13,14^ first proposed two non-traditional layouts: the flying-V layout and the fishbone layout. On this basis, there are the chevron layout^15^, the fishbone triangle layout^16^ and the inverted-V layout^17^. Öztürkoğlu^18^ further proposed the leaf layout and butterfly layout. Based on these layouts, other layouts classified based on the number of aisles and the number of input/output points (I/O points) were further derived. Compared with the flying-V layout and the fishbone layout, the chevron, leaf, and butterfly layouts relaxed the assumption that the aisles must be horizontal or vertical^18^, but at the same time, since aisles can take any angle, the difficulty of implementation is also increased. Therefore, the flying-V layout and fishbone layout are easier to apply in practice. Due to the previous research^19^ on the V-shaped layout, this paper selects the fishbone layout as the research object (Fig. 1).

Class-based storage refers to the classification of goods according to certain standards (volume, turnover rate, etc.). Each class of goods has a fixed storage location, but in the storage area of each class, the assignment of each storage location is random. In this paper, using the common ABC class-based storage, the goods are divided into three classes: A, B and C according to the frequency of goods in and out of the warehouse. At the same time, as shown in Fig. 1, it is divided based on the area proportions. The storage space close to the I/O point of the warehouse is used to store class A, followed by class B and class C. Among them, the proportions could be changed. This paper also uses different area proportions to verify the model in the simulation.

The research motivation of this paper is as follows:

- The warehouse layout design has a significant impact on the walking distance of picking operations^19^, and the fishbone layout with diagonal cross-aisle design can effectively reduce the walking distance of picking operations^20^, possibly the warehouse layout design that can be applied and practiced faster.
- A good storage strategy makes it possible to improve the operational efficiency of the warehouse, and the class-based storage policy is more widely used in practice^19^, but few studies have applied it to non-traditional layout warehouses.
- The comparison of different routing strategies provides more choices for picking operations in the fishbone layout warehouse, and the warehouse can choose the appropriate routing strategy according to its actual operations.

Therefore, this paper will study the routing strategies of the fishbone layout under the class-based storage policy.

The main contributions of this paper are as follows:

- For the fishbone layout under class-based storage, stochastic models of the expected walking distance generated by the S-shape routing strategy and return routing strategy are constructed. Among them, the construction of the stochastic models of the expected walking distance of the two routing strategies in the fishbone layout is newly proposed in our paper.
- This paper innovatively applies the class-based storage policy to the fishbone layout warehouse, which is also a new contribution of this paper.
- Another new contribution of this paper is classifying the fishbone layout warehouse by area through straight-line segmentation, providing a new reference for the application of the class-based storage policy in non-traditional layout warehouses.
- The influence of the S-shape and return routing strategies on the picking walking distance is analyzed.
- By comparing the expected distance of picking travel distance under different ordering frequencies and space allocation, the two routing strategies are evaluated.

**Figure 2:**
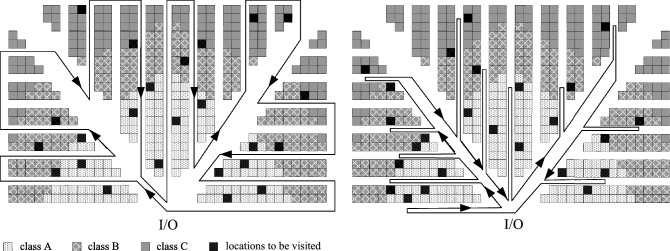
S-shape and return routing strategies in the fishbone layout warehouse under the class-based storage strategy (created with Microsoft Visio Professional 2019, 16.0.14827.20216, https://www.microsoft.com/en-gb/microsoft-365/visio/flowchart-software).

The remainder of this article is organized as follows: the next section presents the related literature. Section “Stochastic models of routing strategies in fishbone layout” studies the ABC class-based storage of fishbone layout warehouses, and constructs the stochastic models of expected walking distance for the S-shape routing strategy and the return routing strategy. In section “Simulation and verification”, the two routing strategies are verified by simulation using MATLAB, and their performances are evaluated. Section “Conclusions and future research” sorts out and summarizes the work of the full text, and points out direction of follow-up research. The S-shape routing strategy and return routing strategy of the fishbone warehouse layout under the class-based storage strategy are shown in Fig. 2.

## Related literature

For the research on non-traditional warehouse layouts, in addition to the aforementioned content, based on the flying-V layout and fishbone layout^13,14^, Pohl et al.^21^ further elaborated the dual-command picking operations in the fishbone layout warehouse. Hall^22^designed a warehouse with two cross aisles to shorten the route length. It is proven that layout setting is one of the key factors affecting the efficiency of picking operations. Cardona et al.^20^ established a nonlinear mathematical model to explore the influence of the angle of the diagonal cross-aisle in the fishbone layout warehouse on the routing strategy, obtained the optimal angle of the diagonal cross-aisle to minimize the expected distance required for order picking, and further elaborated the three-dimensional detailed design of fishbone layouts^23^. Çelik and Süral^10^ studied order picking in traditional parallel-aisle and fishbone layout warehouses from the perspective of different batch sizes and demand skewness levels of orders. Through simulation verification, it was found that the fewer the kinds of items ordered, the greater the difference in average walking time between the traditional layout and the fishbone layout warehouses. Zhou et al.^24^ found that in warehouses with the fishbone layout, the walking distance for order picking is expected to be smaller than in warehouses with the traditional layout, but at the same time, more storage space needs to be added to ensure that storage area requirements are met. Bortolini et al.^25^ designed a V-shaped layout with straight diagonal cross-aisles and verified it with single-command operations, which can save 7–17% of the picking travel distance compared with that of the flying-V layout. Öztürkoğlu et al.^26^ proposed optimal designs for the chevron, leaf and butterfly layouts, and verified them by simulation. Of them, the chevron layout is consistent with the research results of Meller^15^. In the single-command picking operation under the random storage strategy, the chevron warehouse layout has more practical value. Furthermore, alternative aisle designs with multiple pickup and deposit (P&D) points^1^, leverage design^27^, leaf layout with multiple P&D points^28^, and a discrete cross-aisle design^29^ have been proposed. Mesa^30^ designed the diamond layout and the modified diamond warehouse layout based on the fishbone layout and flying-V layout. Based on the research results of Accorsi et al.^31^ on the application of 2- and 3-class-based storage strategies in a V-shaped layout, Bortolini et al.^32^ designed a four-aisle V-shaped layout under the class-based storage strategy. Chae et al.^33^ proposed a double row layout to minimize the total amount of material moved. Kocaman et al.^34^ investigated the best layouts with arbitrary angles of picking and crossing aisles in a continuous space for single-command travel. Ozden et al.^35^ proposed an open-source warehouse layout system that automatically generates and evaluates layout designs for warehouses, using the average walking distance of the picker as the objective function.

Non-traditional layouts provide new ideas and methods for the rational design of warehouse layouts. At present, the common storage strategies are random storage, dedicated storage, volume-based storage, class-based storage and mixed-shelves storage^9,36–44^. Among them, the class-based storage strategy is the most widely used, and many scholars have performed extensive research on it^19^. Lee et al.^45^ studied the storage capacity of a warehouse under the dedicated storage policy to reduce the cost of storage space. Graves et al.^46^ found that there is a positive correlation between warehouse storage space and the number of item kinds, and class-based storage has a greater demand for storage space than random storage. Le-Duc and De Koster^47^ reached the same conclusion through modeling and simulation. Petersen et al. proposed volume-based storage^36^ and proved that this policy is superior to the random storage policy^37^. Petersen et al. also compared the class-based, volume-based, and random storage strategies, and simulation results showed that class-based storage is better than random storage, and approaches volume-based storage^38^. They found that batching orders can save the greatest cost when the order size is small, while class-based and volume-based storage policies result in nearly the same cost savings as batching, which are not affected by order size^39^. Van den Berg^48^ studied the impacts of random storage and class-based storage on the picking operation of an automated storage/retrieval system (AS/RS). Adil et al.^49^ found that, compared with dedicated policy, class-based storage requires less storage space. Zhou et al.^50^ improved the class-based storage strategy through an association algorithm and proved that the improved class-based storage strategy is better than the traditional ABC class-based storage strategy. Furthermore, De Koster et al.^9^, Rao et al.^51^, Žulj et al.^43^ have also studied storage strategies for the traditional layout, while Pohl et al.^52^, Çelik et al.^10^, Venkitasubramony et al.^53^, Bortolini et al.^32^, and Accorsi et al.^31^ have done so for non-traditional layouts.

In terms of the routing strategy, common routing strategies include the S-shape strategy, the return strategy, the midpoint strategy, the largest gap strategy, and the aisle-by-aisle strategy^10,54–56^. These routing strategies are applicable to the storage area of the single warehouse. Roodbergen and De Koster^57^ proposed an improved method, which can be applied to the picking of multiple-block warehouses improving picking efficiency. Petersen^58^ compared the composite, largest gap, midpoint, return, transversal, and optimal routing methods, and concluded that the largest gap and optimal routing policies are better than simple heuristic strategies. Roodbergen and Vis^59^ determined the optimal number of aisles in a warehouse by establishing a nonlinear programming model for the average walking distance of picking operations. Helsgaun et al.^60^ provided LKH (Lin-Kernighan-Heuristic) for the traveling salesman problem (TSP) to generate optimal or near-optimal results. Christophe et al.^61^ compared the LKH algorithm with dedicated S-shape, largest gap, combined and aisle-by-aisle heuristics, and concluded that LKH saves 47% more of the route distance than the latter. Hong et al.^62^ established an exponential batch model by using the mixed integer programming method and solved the model by using the simulated annealing algorithm, which reduced the search time in the case of storage congestion. Battini et al.^63^ proposed the joint strategy of storage assignment and travel distance of order pickers to achieve the goal of optimizing the order picking system. Zhou et al.^19,64^ established stochastic models for the S-shape and return routing strategies of the traditional layout and V-type layout under class-based storage, respectively, and obtained the applicability of the two routing strategies. Zhu et al.^65^ assumed that the arrival of orders obeyed the Poisson, established stochastic models of S-shape and return routing strategies under the class-based storage policy, and identified the applicable cases of the two routing strategies. Ozden et al.^66^ introduced the visibility graph for the distance of the picking travel, and experiments proved that using the visibility graph to solve the results is more accurate. Moreover, Öztürkoğlu^67^, Shavaki et al.^68^ have used heuristic algorithms to study routing strategies, Bottani et al.^69^, Kulak et al.^70^, Bodis et al.^71^, Sebo et al.^72^ employed meta-heuristics, and Dijkstra et al.^73^, Masae et al.^55,56,74^ applied exact algorithms. There are also studies on other order picking strategies, such as dynamic picking^75^.

By summarizing and sorting out the existing literature, as shown in Table 1, the following can be found:

- In addition to the traditional warehouse layout, the existing research on non-traditional layouts mainly includes the V-shaped layout, fishbone layout, chevron layout and leaf layout, among which most focus on the V-shaped and fishbone layouts. Moreover, these two layouts are easier to apply and practice due to the complex and varied angles of shelves in the chevron layout and the leaf layout.
- Existing storage strategies mainly include random storage, dedicated storage, volume-based storage, class-based storage and mixed-shelves storage^9,36–44^. In the actual production practice of manual warehouses, the class-based storage strategy and random storage strategy are widely used, and are mostly for traditional layouts. About the fishbone layout, most of them focus on random storage, lack of research on fishbone layout warehouses under the class-based storage strategy.
- The existing research on the stochastic model of the walking distance of the picking operation is mostly aimed at the traditional layout and V-shape layout, and mainly focuses on single-command or dual-command operations, and there is little research on picking tours in fishbone layout warehouses. Few studies have been carried out on the stochastic model of picking travel distance in the fishbone layout under the class-based storage strategy.

Therefore, this paper: (1) selects the fishbone layout that has been more applied and practiced as the research object to enrich the theoretical basis for its practice; (2) innovatively applies the class-based storage strategy to the fishbone layout warehouse, supplementing the existing literature on the fishbone layout warehouse under the class-based storage policy; (3) constructs stochastic models of S-shape and return routing strategies in the fishbone layout, and compares the performance of the two picking routing strategies based on the class-based storage strategy, filling the research on picking tours in the fishbone layout warehouse.

**Table 1:** Comparison with the existing literature.

References	Research points	Warehouse layout	Storage policy	Routing strategy	Model/Method
Zhou et al.^19^	Routing strategy	V-shaped	Class-based	S-shape, return	Stochastic model of the walking distance
Cardona et al.^23^	Warehouse layout	3D fishbone	Random	Single-command	An integer nonlinear optimization model
Öztürkoğlu et al.^26^	Warehouse layout	Chevron, leaf, butterfly	Random	Single-command	Expected travel distances
Accorsi et al.^31^	Storage policy	V-shaped	Class-based	Single-command	Access time model
Bortolini et al.^32^	Storage policy, and warehouse layout	Four-aisle V-shaped	Class-based	Single-command	Analytic model
Petersen et al.^38^	Storage policy	Traditional	Class-based, volume-based, random	Optimal	Warehouse simulation model
Masae et al.^55^	Routing strategy	Chevron	Random, turnover-based	Optimal, midpoint, largest gap, S-shape	Graph theory and dynamic programming
Masae et al.^56^	Routing strategy	Leaf	Random, turnover-based	Optimal, S-shape, return, midpoint, largest gap	Eulerian graph and dynamic programming
Zhou et al.^64^	Routing strategy	Two blocks	Class-based	S-shape, return	Stochastic model of the walking distance
Zhu et al.^65^	System efficiency	Two blocks	Class-based	S-shape, return	Stochastic model of the walking distance
Masae et al.^74^	Routing strategy	Two blocks		Optimal, S*-shape	Graph theory and dynamic programming
This work	Storage policy, and routing strategy	Fishbone	Class-based	S-shape, return	Stochastic model of the walking distance

**Figure 3:**
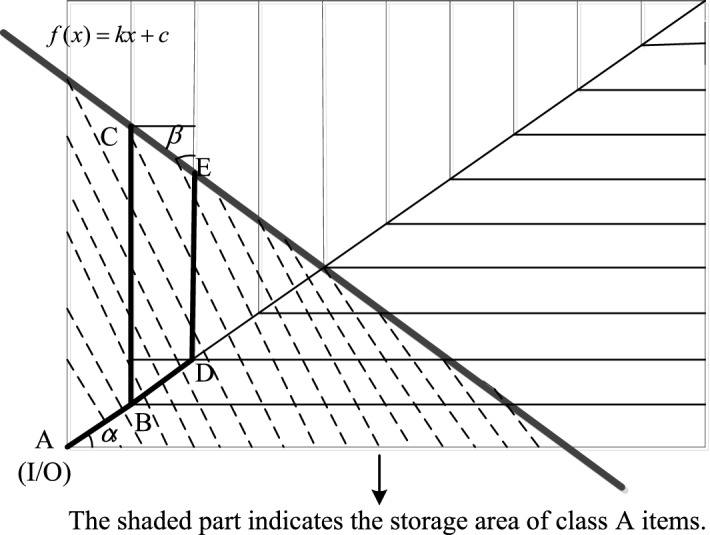
ABC class-based storage strategy in the fishbone layout warehouse.

## Stochastic models of routing strategies in fishbone layout warehouses

In warehouses with traditional manual picking operations, the main factors affecting operational efficiency include the storage strategy, warehouse layout and routing strategy. In this section, the storage areas of classes A, B and C items will be divided according to the proportional linear to meet the classification requirements of the three classes of items. Then, stochastic models of the expected walking distance for the S-shape routing strategy and return routing strategy in the fishbone layout under the class-based storage strategy are established to lay the foundation for the simulation and verification.

For the convenience of research, it is assumed that the warehouse will synthesize all orders received into one picking list within a certain period. The items required by orders can be obtained at a specific location in the warehouse, and can meet the quantity requirements of orders. Because the arrival of the orders is random, the items that need to be picked are also random. In the warehouse, the probability of all goods is estimated according to the historical actual data, and goods are classified and stored according to the frequency to be picked. There is only one I/O point in the middle of the front aisle, and the left half-warehouse and right half-warehouse are symmetrical. And the storage of each area of each class is random. Therefore, only the right half-warehouse is considered in this paper.

### ABC class-based storage strategy of the fishbone layout

The class-based storage strategy studied in this paper is based on the area proportion, and the storage area close to the I/O point of the warehouse is for class A items, followed by class B and class C. Among them, the area proportions of classes A, B and C in the total warehouse can be changed.

As shown in Fig. 3, in the right half-warehouse, according to the design principle that class A goods are closest to the I/O point, two straight lines are adopted. According to the characteristics of the fishbone layout, the storage area of class A goods is intercepted, and the distances between the I/O point and the most edge of class A goods are equal. The symbols used in the model are defined as follows: $l_1$ is the aisle width; $l_2$ is the width of the double shelf; *a* is half the length of the warehouse; *b* is the width of the warehouse; $\alpha$ is the angle of the diagonal cross-aisle; and $\alpha _0$ is the diagonal angle of the right half-warehouse.

In Fig. 3, point A is the I/O point of the fishbone layout, and points C and E are the farthest storage locations in the aisles of class A items. Line segments BC and DE are the picking aisles in the fishbone layout, and BD is the diagonal cross-aisle. In the right half-warehouse of the fishbone layout, the straight line $f(x) = kx + c$ is used to intercept the storage area of Class A items, and BC=BD+DE. According to this equation, the slope *k* and intercept *c* of line *f*(*x*) are determined.

1 $$\begin{aligned} \tan \beta = \frac{l}{\frac{l}{\cos \alpha }-l\cdot \tan \alpha }, \end{aligned}$$

among them, $$l = {l_1} + {l_2}$$.

Thus, the value of slope *k* of the straight line $f(x) = kx + c$ is:

2 $$\begin{aligned} k=\tan \left( \frac{\pi }{2}+\arctan \beta \right) . \end{aligned}$$

When the split ratio of class A items’ area $P_A>-\frac{b}{ak}$,

3 $$\begin{aligned} c = \sqrt{ - 2k\left( ab - ab{P_A}\right) } - ak + b, {P_A}=\frac{{S_A}}{S} = \frac{{\frac{1}{2}}\cdot c\cdot ({\frac{c}{-k})}}{ab}, \end{aligned}$$

where ${S_A}$ is the area for class A items in the fishbone layout.

Similarly, when $${P_A} \le - \frac{a}{2bk}$$,

4 $$\begin{aligned} c = \sqrt{ - 2ab{P_A}k} , \end{aligned}$$

when $$- \frac{a}{2bk}< {P_A} < - \frac{a}{bk}$$,

5 $$\begin{aligned} c = \frac{b - 2kb{P_A}}{2}. \end{aligned}$$

Similarly, the intercept of class B items and class C items can be obtained.

### Model assumptions and notations

**Figure 4:**
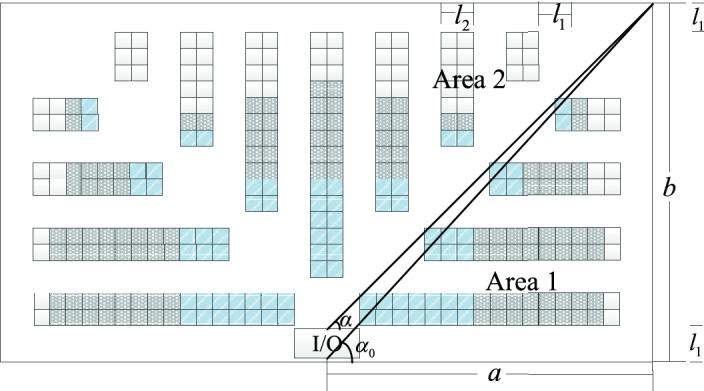
Fishbone layout under the class-based storage strategy.

Under the class-based storage policy, goods are grouped into *M* classes according to the frequency in and out of the warehouse, and in descending order according to the distances to the I/O point of the warehouse. The goods are placed in corresponding positions. In each class area, items are stored randomly. As shown in Fig. 4, the goods are grouped into classes A, B and C (fast, medium and slow), and the right half-warehouse is divided into two areas, in which the lower part is Area 1, and the upper part is Area 2. The numbers of aisles in Area 1 and Area 2 are $n_1$ and $n_2$. The descriptions of all notations are shown in Table 2.

**Table 2:** Descriptions of notations.

Notations	Descriptions
*a*	The length of the right half-warehouse
*b*	The width of the warehouse
${l_1}$, ${l_2}$	The widths of the aisles and double shelves
${\alpha _0}$	The diagonal angle of the right half-warehouse
$\alpha$	The angle of the diagonal cross-aisle
*S*	The area of the fishbone layout warehouse
${S_A}$, ${S_B}$, ${S_C}$	The areas of class A, class B and class C items in the fishbone layout warehouse
*k*	The slope of the line *f*(*x*)
*c*	The intercept of the line *f*(*x*)
${P_A}$	The split ratio of class A items’ area
${n_1}$, ${n_2}$	The numbers of aisles in Area 1 and Area 2
${m_{aj}}~$, ${m_{bj}}~$, ${m_{cj}}~$	The lengths of that class A, class B and class C items account in the *j*th picking aisle
${p_a}$, ${p_b}$, ${p_c}$	The picking probabilities of class A, class B and class C items
${p_{aj}}~$, ${p_{bj}}~$, ${p_{cj}}~$	The picking probabilities of class A, class B and class C items in the *j*th aisle
${T_j}$	The number of item kinds expected to be picked in the *j*th aisle
$p_{aj}^{(K)}$, $p_{bj}^{(K)}$, $p_{cj}^{(K)}$	The probabilities of *K* item kinds among the *T* item kinds in the storage areas of class A, class B and class C in the *j*th aisle
$d_{\mathrm{{a}}j}^{(K)}$	The expected maximum distance required to pick class A items in the *j*th aisle
${d_{aj}}(T)$	The expected walking distance in the *j*th aisle when picking *T* kinds of goods in one order
${p_j}$	The probability of goods picked in the *j*th aisle
${R_{1far}}~$, ${R_{2far}}$	The picking travel distances generated by ${\bar{j}_{1far}}$ in Area 1 and ${\bar{j}_{2far}}$ in Area 2
${j_{1far}}~$, ${j_{2far}}$	The farthest picking aisles that have picking operations in Area 1 and Area 2
${\bar{j}_{1far}}~$, ${\bar{j}_{2far}}$	The expected farthest aisles with goods picked in Area 1 and Area 2
${D_{return}}~~(T)$	The picking travel distance of the return routing strategy in the right half-warehouse
$p_{aj}^ \wedge$, $p_{bj}^ \wedge$, $p_{cj}^ \wedge$	The probabilities of at least one kind of goods in storage areas of class A, class B and class C in the *j*th aisle
${d_{aj}}$, ${d_{bj}}$, ${d_{cj}}$	The expected walking distances for picking class A, class B and class C items in the *j*th aisle
${d_j}$	The expected walking distance in the *j*th aisle of the S-shape routing strategy
${p_{1{j_{_1far}}}}~~$, ${p_{2{j_{_2far}}}}$	The probabilities of the farthest aisles that have picking operations in Area 1 and Area 2 when picking *T* items
${D_S}(T)$	The walking distance of the S-shape routing strategy in the right half-warehouse
${p_{1odd}}~$, ${p_{2odd}}$	The probabilities when the number of aisles for picking goods in Area 1 or Area 2 is odd
${m_{{j_1}}}$, ${m_{{j_2}}}$	The additional distances in Area 1 and Area 2 of the S-shape routing strategy

In order to construct the stochastic models of the routing strategies, we make the following assumptions.

- The warehouse has only one I/O point on the middle of the front aisle, that is, the left half-warehouse and the right half-warehouse are symmetrical about the centerline. Since items in each class area are stored randomly, only the right half-warehouse is considered in this paper.
- The storage areas are determined according to the length of the shelf, and the height of the shelf is not considered.
- When picking in the aisle, the goods on the shelves on both sides can be picked, and the distance to the two sides can be ignored.
- The picking items are random on demand and independent of each other in orders.
- For order picking, the probability of picking each item of each kind (not class) is the same.
- Goods of the same kind are randomly assigned into one storage location and stored in only one storage location.
- The influence of warehouse height on warehouse layout is ignored.
- In a certain aisle, items of each class on the shelf are evenly distributed.
- The main aisles and picking aisles are equal in width.

The angle of the main aisle in the fishbone layout varies from 0° to 90°. Therefore, the numbers of aisles in Area 1 and Area 2 are:

when $$\arctan (\frac{{l_2}/2}{a - {l_1}/2\sin \alpha }) \le a < \alpha _0$$:

6 $$\begin{aligned} {n_1} = \left\lfloor {\frac{{\left( a - \frac{{l_1}}{2\sin \alpha } -{l_1}\right) \tan \alpha - 0.5{l_2}}}{{{l_1} + {l_2}}}} \right\rfloor ,{n_2} = \left\lfloor {\frac{{a - {l_1}}}{{{l_1} + {l_2}}}} \right\rfloor , \end{aligned}$$

when $$\alpha _0 \le a < \pi /2$$:

7 $$\begin{aligned} {n_1} = \left\lfloor {\frac{{b - 2{l_1} - 0.5{l_2}}}{{{l_1} + {l_2}}}} \right\rfloor ,{n_2} = \left\lfloor {\frac{{\left( b - \frac{{l_1}}{2\cos \alpha }-2{l_1}\right) /\tan \alpha }}{{{l_1} + {l_2}}}} \right\rfloor . \end{aligned}$$

### Stochastic model of the return routing strategy

The walking route of the return routing strategy is as follows: the picker enters from one end of the picking aisle, first picks the items required by the order on one side along the direction of the picking aisle, returns the same way after completing the picking operation of the items to be picked farthest from the aisle, and then picks the items to be picked on the other side until they return to the aisle and enter the next picking aisle.

The return routing strategy of the fishbone layout is shown as the right sub-figure in Fig. 2. The walking distance of the return routing strategy for picking mainly includes three parts: one is the expected distance that is generated by the farthest goods’ location that needs to be picked in each picking aisle; the second is the distance from the diagonal cross-aisle to the picking aisle; and the third is the walking distance in the diagonal cross-aisle that is generated by the farthest picking aisle with goods to be picked.

The length of class A items in the *j*th picking aisle is:

8 $$\begin{aligned} {m_{\mathrm{{a}}j}} = \max \left( 0,\min \left( a,ca + \left( {l_1} + {l_2}\right) \cdot \left( j - 0.5\right) \cdot \tan \left( 90 + \arctan \cos \alpha \right) - \left( {l_1} + {l_2}\right) \cdot \left( j - 0.5\right) \cdot \tan \alpha \right) \right) . \end{aligned}$$

The length of class B items in the *j*th picking aisle is:

9 $$\begin{aligned} {m_{bj}} = \max \left( 0,\min \left( a,cb + \left( {l_1} + {l_2}\right) \cdot \left( j - 0.5\right) \cdot \tan \left( 90 + \arctan \cos \alpha \right) - \left( {l_1} + {l_2}\right) \cdot \left( j - 0.5\right) \cdot \tan \alpha \right) \right) . \end{aligned}$$

The length of class C items in the *j*th picking aisle is:

10 $$\begin{aligned} {m_{\mathrm{{c}}j}} = {a} - {m_{bj}} - {m_{aj}} - \left( {l_1} + {l_2}\right) \cdot \left( j - 0.5\right) \cdot \tan \alpha . \end{aligned}$$

The picking probability of class A items in the *j*th picking aisle is:

11 $$\begin{aligned} {p_{aj}} = {p_a} \cdot {\frac{{m_{aj}}}{{\sum \limits _{j = 1}^{{n_1} + {n_2}} {{m_{aj}}} }}},\mathrm{{ }}j = 1,2, \cdots , {n_1}\mathrm{{ + }}{n_2} , \end{aligned}$$

where ${p_a}$ is the picking probability of class A items.

The picking probability of class B items in the *j*th picking aisle is:

12 $$\begin{aligned} {p_{bj}} = {p_b} \cdot \frac{{{m_{bj}}}}{{\sum \limits _{j = 1}^{{n_1} + {n_2}} {{m_{bj}}} }},\mathrm{{ }}j = 1,2, \cdots , {n_1}\mathrm{{ + }}{n_2} , \end{aligned}$$

where ${p_b}$ is the picking probability of class B items.

The picking probability of class C items in the *j*th picking aisle is:

13 $$\begin{aligned} {p_{cj}} = {p_c} \cdot \frac{{m_{cj}}}{{\sum \limits _{j = 1}^{{n_1} + {n_2}} {{m_{cj}}} }},\mathrm{{ }}j = 1,2, \cdots , {n_1}\mathrm{{ + }}{n_2} , \end{aligned}$$

where ${p_c}$ is the picking probability of class C items.

Of them, for the *j*th aisle in the right half-warehouse, the kinds of items to be picked on the shelves classified as A, B and C obey the binomial distribution *b*(*K*; *T*). Then, we assumed that the kinds of items expected to be picked in the *j*th aisle is $T_j$, so that the probability of *K* kinds among the *T* kinds in the class A storage area in the *j*th aisle is $p_{aj}^{(K)}$, so there are:

14 $$\begin{aligned} p_{aj}^{(K)} = \mathrm{{C}}_T^K{(1 - {p_{aj}})^{T - K}}{({p_{aj}})^K},\mathrm{{ }}K = 0,1, \cdots ,T;1 \le j \le {n_1} + {n_2}. \end{aligned}$$

Similarly, the probability of *K* kinds among *T* kinds of goods in class B storage area in the *j*th aisle is:

15 $$\begin{aligned} p_{bj}^{(K)} = \mathrm{{C}}_T^K{\left( 1 - {p_{bj}}\right) ^{T - K}}{\left( {p_{bj}}\right) ^K},\mathrm{{ }}K = 0,1, \cdots ,T;1 \le j \le {n_1} + {n_2}. \end{aligned}$$

The probability of *K* kinds among *T* kinds of goods in class C storage area in the *j*th aisle is:

16 $$\begin{aligned} p_{cj}^{(K)} = \mathrm{{C}}_T^K{(1 - {p_{cj}})^{T - K}}{({p_{cj}})^K},\mathrm{{ }}K = 0,1, \cdots ,T;1 \le j \le {n_1} + {n_2}. \end{aligned}$$

It is assumed that there are *K* kinds of class A items to be picked for orders in the *j*th aisle, the expected maximum distance required to pick class A items in this aisle is $d_{\mathrm{{a}}j}^{(K)}$, and the items in the storage area of class A in the *j*th aisle are evenly distributed with a uniform distribution:

17 $$\begin{aligned} d_{aj}^{(K)} = \mathrm{{E}}\left( \max \left( {\xi _{a1}},{\xi _{a2}}, \cdots, {\xi _{aK}}\right) \right) . \end{aligned}$$

The distribution function of $\max ({\xi _{a1}},{\xi _{a2}}, \cdots, {\xi _{aK}})$ is:

18 $$\begin{aligned} F(x) = p\{ \max \left( {\xi _{a1}},{\xi _{a2}}, \cdots ,{\xi _{aK}}\right)< x\} = p\{ {\xi _{a1}}< x,{\xi _{a2}}< x, \cdots ,{\xi _{aK}} < x\} = \frac{{{x^K}}}{{m_{aj}^K}}, 0 \le x \le {m_{aj}}. \end{aligned}$$

Then,

$$\begin{aligned} \mathrm{{ }}\mathrm{{E}}\left( \max \left( {\xi _{a1}},{\xi _{a2}}, \cdots ,{\xi _{aK}}\right) \right) = \int _0^{{m_{aj}}} {x\mathrm{{d}}\left( \frac{{{x^K}}}{{m_{aj}^K}}\right) } = \frac{K}{{K + 1}}{m_{aj}}, \end{aligned}$$

19 $$\begin{aligned} d_{aj}^{(K)} = {\frac{K}{{K + 1}}}{m_{aj}}. \end{aligned}$$

Therefore, when picking *T* kinds of goods in one order, the expected walking distance in the *j*th aisle is:

20 $$\begin{aligned} {d_{aj}}(T) = \mathrm{{E}}\left( d_{aj}^K\right) = \sum \limits _{K = 0}^T {p_{aj}^{(K)}} d_{aj}^{(K)},\mathrm{{ }}~j = 1,2, \cdots ,n_1 + n_2. \end{aligned}$$

The probability of goods picked in the *j*th aisle is:

21 $$\begin{aligned} {p_j} = \frac{{p_{aj}} + {p_{bj}} + {p_{cj}}}{{p_a} + {p_b} + {p_c}}. \end{aligned}$$

Because the numbers of aisles in Area 1 and Area 2 are different, it is necessary to calculate the expected walking distances in the diagonal cross-aisle for Area 1 and Area 2 separately. Recall that when picking *T* items, the probability of the farthest visited aisle ${j_{1far}}$ of Area 1 is ${p_{1{j_{_1far}}}}~$; then,

22 $$\begin{aligned} \left\{ \begin{array}{l} {p_{1{j_{1far}}}} = {({p_{1{j_{1far}}}}~)^T},\mathrm{{ }}{j_{1far}} = 1\\ {p_{1{j_{1far}}}} = {\left( \sum \limits _{{j_1} = 1}^{{j_{1far}}} {{p_{1{j_1}}}} \right) ^{T - 1}} \times ({p_{1{j_{1far}}}}~),\mathrm{{ }}2 \le {j_{1far}} \le {n_1} \end{array} \right. . \end{aligned}$$

Therefore, the expected farthest aisle with goods picked in Area 1 is:

23 $$\begin{aligned} {\bar{j}_{1far}} = \mathrm{{E}} \left( {j_{1far}}\right) = \sum \limits _{{j_{1far}}~~= 1}^{{n_1}} {j_{1far}} \times p_{1}{j}_{1far}\Bigg /\sum \limits _{j = 1}^{{n_1}} {{p_{1j}}}. \end{aligned}$$

Then, it can be obtained that the travel distance in the diagonal cross-aisle generated by ${\bar{j}_{1far}}$ is

24 $$\begin{aligned} {R_{1far}} = \left( {\bar{j}_{1far}} - 0.5\right) \times \frac{{l_1} + {l_2}}{\cos \alpha }. \end{aligned}$$

Similarly, remembering that when picking *T* items, the probability of the farthest visited aisle ${j_{2far}}$ in Area 2 is ${p_{2{j_{_2far}}}}~$, then,

25 $$\begin{aligned} \left\{ \begin{array}{l} {p_{2{j_{2far}}}} = {({p_{2{j_{2far}}}}~)^T}, {j_{2far}} = 1\\ {p_{2{j_{2far}}}} = {\left( \sum \limits _{{j_2} = 1}^{{j_{2far}}} {{p_{2{j_2}}}} \right) ^{T - 1}} \times ({p_{2{j_{2far}}}}~),\mathrm{{ }}2 \le {j_{2far}} \le {n_2} \end{array} \right. . \end{aligned}$$

Then, the expected farthest aisle with goods picked in Area 2 is:

26 $$\begin{aligned} {\bar{j}_{2far}} = \mathrm{{E}}\left( {j_{2far}}\right) = \sum \limits _{{j_{2far}}~~= 1}^{{n_2}} {j_{2far}} \times p_{2}{{j}_{2far}}\Bigg /\sum \limits _{j = 1}^{{n_2}} {{p_{2j}}}. \end{aligned}$$

The travel distance in the diagonal cross-aisle generated by ${\bar{j}_{2far}}$ is obtained as follows:

27 $$\begin{aligned} {R_{2far}} = (\bar{j}_{2far} - 0.5) \times \frac{{l_1} + {l_2}}{\sin \alpha }. \end{aligned}$$

Therefore, the picking travel distance of the return routing strategy in the right half-warehouse is:

28 $$\begin{aligned} {D_{return}}(T) = (2 \times \max \left( {R_{1far}},{R_{2far}}\right) + \sum \limits _{j = 1}^{n_1 + n_2} {{d_{aj}}(T) + } \sum \limits _{j = 1}^{n_1 + n_2} {{d_{bj}}(T) + \sum \limits _{j = 1}^{n_1 + n_2} {{d_{cj}}(T)} )} . \end{aligned}$$

### Stochastic model of the S-shape routing strategy

Compared with the return routing strategy, the S-shape routing strategy has its own characteristics. When adopting the S-shape routing strategy, the picker enters from one end of the picking aisle, in which the picker can pick the goods stored on the shelves on both sides of the picking aisle at the same time, and finally exits from the other end of the picking aisle. Meanwhile, the picker will traverse all of the aisles in which some items need to be picked until the picking operation in the last aisle where the goods to be picked is completed, and then return from the last aisle to the I/O point of the warehouse. Moreover, two situations require special attention. If the number of picking aisles that need to complete picking operations is even, the picker returns to the I/O point according to the S-shape routing after completing picking in the last picking aisle, otherwise, the picker returns to the I/O point according to the return routing.

Due to the characteristics of the S-shape routing strategy, the walking distance in the picking aisle where goods are picked is the length of the picking aisle. The S-shape routing strategy in the fishbone layout is shown in the left sub-figure in Fig. 2. The walking distance of the S-shape routing strategy for picking mainly includes three parts: the first is the expected length of the picking aisle where items are picked; the second is the distances from the diagonal cross-aisle, the back aisle and the right aisle to the picking aisle; and the third is the walking distance in the diagonal cross-aisle generated by the farthest picking aisle with goods picked.

Of them, for the *j*th aisle in the right half-warehouse, there are only two cases of “goods picking” and “no goods picking” on the shelves of classes A, B and C. In the case of “no goods picking”, there is no need to enter the picking aisle, and there will be no walking distance. In the case of “goods picking”, as long as there is an item to be picked in the aisle, pickers need to travel the whole aisle. Therefore, only the probability that there is at least one item to be picked in the aisle is considered. It is assumed that there are *T* kinds of goods in the order, and the probabilities $p_{aj}^{\wedge }$, $p_{bj}^ \wedge$ and $p_{cj}^ \wedge$ of at least one kind of good in class A, class B and class C storage areas in the *j*th aisle are:

29 $$\begin{aligned}&p_{aj}^ \wedge = \left[ {1 - {{(1 - {p_{aj}})}^T}} \right] ,\mathrm{{ }}1 \le j \le {n_1} + {n_2}, \end{aligned}$$

30 $$\begin{aligned}&p_{bj}^ \wedge = \left[ {1 - {{(1 - {p_{bj}})}^T}} \right] ,\mathrm{{ }}1 \le j \le {n_1} + {n_2}, \end{aligned}$$

31 $$\begin{aligned}&p_{cj}^ \wedge = \left[ {1 - {{(1 - {p_{cj}})}^T}} \right] ,\mathrm{{ }}1 \le j \le {n_1} + {n_2}, \end{aligned}$$

where $p_{aj}$ is the same as equation (11), $p_{bj}$ is the same as equation (12), and $p_{cj}$ is the same equation (13).

The expected walking distance for picking class A, class B and class C items in the *j*th aisle is:

32 $$\begin{aligned}&{d_{aj}} = \mathrm{{E}}\left( {d_{aj}}\right) = \sum \limits _{j = 0}^{{n_1} + {n_2}} {\left( {p_{aj}^ \wedge \cdot {m_{aj}}} \right) } ,\mathrm{{ }}j = 1,2, \cdots , {n_1}\mathrm{{ + }}{n_2}, \end{aligned}$$

33 $$\begin{aligned}&{d_{bj}} = \mathrm{{E}}\left( {d_{bj}}\right) = \sum \limits _{j = 0}^{{n_1} + {n_2}} {\left( {p_{bj}^ \wedge \cdot {m_{bj}}} \right) } ,\mathrm{{ }}j = 1,2, \cdots , {n_1}\mathrm{{ + }}{n_2}, \end{aligned}$$

34 $$\begin{aligned}&{d_{cj}} = \mathrm{{E}}\left( {d_{cj}}\right) = \sum \limits _{j = 0}^{{n_1} + {n_2}} {\left( {p_{cj}^ \wedge \cdot {m_{cj}}} \right) } ,\mathrm{{ }}j = 1,2, \cdots , {n_1}\mathrm{{ + }}{n_2}. \end{aligned}$$

The expected picking distance in the *j*th aisle is:

35 $$\begin{aligned} {d_j} = \mathrm{{E}}\left( {d_{aj}}\right) \mathrm{{ + }}\mathrm{{E}}\left( {d_{bj}}\right) + \mathrm{{E}}\left( {d_{cj}}\right) . \end{aligned}$$

Because the numbers of aisles in Area 1 and Area 2 are different, it is necessary to calculate the expected walking distance in the diagonal cross-aisle for Area 1 and Area 2 separately. Recall that when picking *T* items, the probability of the farthest visited aisle ${j_{1far}}$ of Area 1 is ${p_{1{j_{_1far}}}}~$; then,

36 $$\begin{aligned} \left\{ \begin{array}{l} {p_{1{j_{1far}}}} = {({p_{1{j_{1far}}}}~)^T}, \ {j_{1far}} = 1\\ {p_{1{j_{1far}}}} = {\left( \sum \limits _{{j_1} = 1}^{{j_{1far}}} {{p_{1{j_1}}}} \right) ^{T - 1}} \cdot ({p_{1{j_{1far}}}}~),\mathrm{{ }}2 \le {j_{1far}} \le {n_1} \end{array} \right. . \end{aligned}$$

Therefore, the expected farthest aisle with goods picked in Area 1 is:

37 $$\begin{aligned} {\bar{j}_{1far}} = \mathrm{{E}}\left( {j_{1far}}\right) = \sum \limits _{{j_{1far}} ~~= 1}^{{n_1}} {j_{1far}} \cdot p_{1}{j}_{1far}\bigg /\sum \limits _{j = 1}^{{n_1}} {p_{1}{j}} . \end{aligned}$$

In summary, the travel distance in the diagonal cross-aisle generated by the expected farthest aisle in Area 1 with goods picked can be obtained from:

38 $$\begin{aligned} {R_{1far}} = \left( {\bar{j}_{1far}} - 0.5\right) \cdot \frac{{{l_1} + {l_2}}}{{\cos \alpha }}. \end{aligned}$$

Similarly, remembering that when picking *T* items, the probability of the farthest visited aisle ${j_{2far}}$ in Area 2 is ${p_{2{j_{_2far}}}}~$, then,

39 $$\begin{aligned} \left\{ \begin{array}{l} {p_{2{j_{2far}}}} = {({p_{2{j_{2far}}}}~)^T}, {j_{2far}} = 1\\ {p_{2{j_{2far}}}} = {\left( \sum \limits _{{j_2} = 1}^{{j_{2far}}} {{p_{2{j_2}}}} \right) ^{T - 1}} \cdot ({p_{2{j_{2far}}}}~),\mathrm{{ }}2 \le {j_{2far}} \le {n_2} \end{array} \right. . \end{aligned}$$

The expected farthest aisle with goods picked in Area 2 is:

40 $$\begin{aligned} {\bar{j}_{2far}} = \mathrm{{E}}\left( {j_{2far}}\right) = \sum \limits _{{j_{2far}}~~= 1}^{{n_2}} {j_{2far}} \cdot p_{2}{j_{2far}} \bigg /\sum \limits _{j = 1}^{{n_2}} {{p_{2j}}}. \end{aligned}$$

The walking distance in the diagonal cross-aisle generated by ${\bar{j}_{2far}}$ is:

41 $$\begin{aligned} {R_{2far}} = \left( {\bar{j}_{2far}} - 0.5\right) \cdot \frac{{{l_1} + {l_2}}}{{\sin \alpha }}. \end{aligned}$$

Therefore, the walking distance of the S-shape routing strategy in the right half-warehouse is:

42 $$\begin{aligned} {D_{S}}(T) = \left[ 2 \cdot \max ({R_{1far}},{R_{2far}}) + \sum \limits _{j = 1}^{n_1 + n_2} {\left( {d_{aj}}(T) + {d_{bj}}(T) + {{d_{cj}}(T)}\right) }\right] + {p_{1odd}} \cdot {m_{{j_1}}} + {p_{2odd}} \cdot {m_{{j_2}}}. \end{aligned}$$

where ${p_{1odd}}$ and ${p_{2odd}}$ represent the probabilities when the numbers of aisles for picking goods in Area 1 and Area 2 are odd, and the additional distances increased are ${m_{{j_1}}}$ and ${m_{{j_2}}}$.

## Simulation and verification

To verify the effect of the stochastic models of the two routing strategies under the class-based storage strategy in the fishbone warehouse layout, the models need to be approximately calculated and compared with the simulation results. According to the ordering frequency and storage space allocation of different kinds of goods, this paper sets five cases. The specific data are shown in Table 3. Referring to the actual data of the distribution center, assuming that the warehouse parameters are $a = 300$, $b = 300$, and $\alpha = 45^\circ$, the widths of the picking aisle and the double shelf are 2, there is only one order at present, and the number of items to be picked in the order is 8, which is the expected value in a single picking operation for a single picker from the actual data of the e-commerce distribution center. Then the differences between the model results and the simulation results, and between the S-shape routing strategy and the return routing strategy will be displayed. All numerical tests are implemented by MATLAB 2020a on a computer with an AMD Ryzen 7 4800U CPU with Radeon graphics.

**Table 3:** Relationship between ordering frequency of item classes and storage space assignment.

Ordering frequency/Space assignment	Class A	Class B	Class C
Case 1	33.33/33.33	33.33/33.33	33.33/33.33
Case 2	45/30	30/30	25/40
Case 3	60/25	25/30	15/45
Case 4	75/20	20/30	5/50
Case 5	85/15	10/30	5/55

In order to facilitate the research, the simulated data are all self-created according to the actual data of an e-commerce distribution center, including the frequency of orders, the types of items, and the allocation of storage areas. Based on the actual data of the e-commerce distribution center, the positions of the ordered items on the shelves in the warehouse are counted. Since the items in the same class have the same ordering frequency, the ordering frequency can be calculated from the empirical distribution of the actual demand for the items over a period of time, which is estimated as a constant based on the actual data, as shown in Table 3. Additionally, the probabilities of the distribution of the classes of items to be picked in the simulation can be simulated based on the location information of the items to be picked contained in the actual data, as shown in Table 4.

**Table 4:** Probabilities of the distribution of the classes of items to be picked in the simulation.

	1	2	3	4	5	6	7	8	Total
**Case 1**									
A	0.34	0.34	0.34	0.34	0.33	0.33	0.33	0.33	2.68
B	0.33	0.33	0.33	0.33	0.34	0.34	0.33	0.33	2.66
C	0.33	0.33	0.33	0.33	0.33	0.33	0.34	0.34	2.66
**Case 2**									
A	0.63	0.63	0.48	0.48	0.08	0.08	0.01	0.01	2.40
B	0.36	0.36	0.48	0.48	0.30	0.30	0.06	0.06	2.40
C	0.01	0.01	0.04	0.04	0.62	0.62	0.93	0.93	3.20
**Case 3**									
A	0.60	0.60	0.35	0.35	0.04	0.04	0.01	0.01	2.00
B	0.38	0.38	0.47	0.47	0.30	0.30	0.05	0.05	2.40
C	0.02	0.02	0.18	0.18	0.66	0.66	0.94	0.94	3.60
**Case 4**									
A	0.60	0.60	0.18	0.18	0.01	0.01	0.01	0.01	1.60
B	0.32	0.32	0.52	0.52	0.31	0.31	0.05	0.05	2.40
C	0.08	0.08	0.30	0.30	0.68	0.68	0.94	0.94	4.00
**Case 5**									
A	0.61	0.61	0.18	0.18	0.02	0.02	0.01	0.01	1.64
B	0.29	0.29	0.48	0.48	0.20	0.20	0.03	0.03	2.00
C	0.10	0.10	0.34	0.34	0.78	0.78	0.96	0.96	4.36

For simulation experiments, the number of simulations needs to be determined, so that the relative error between the simulation and the actual average walking distance converge to an acceptable small range under a certain confidence level. Referring to Law and Kelton^76^, we perform 10,000 simulations on each order, which fully meets the relative error requirements. The specific simulation steps and pseudo-code are shown in Algorithm 1.
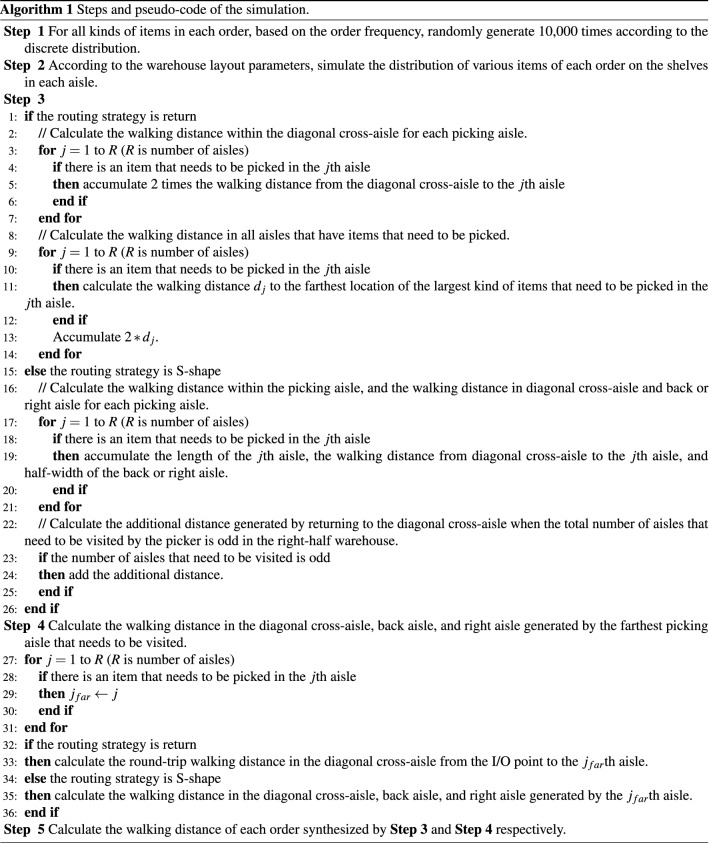


### Simulation and validation of the return routing strategy

**Figure 5:**
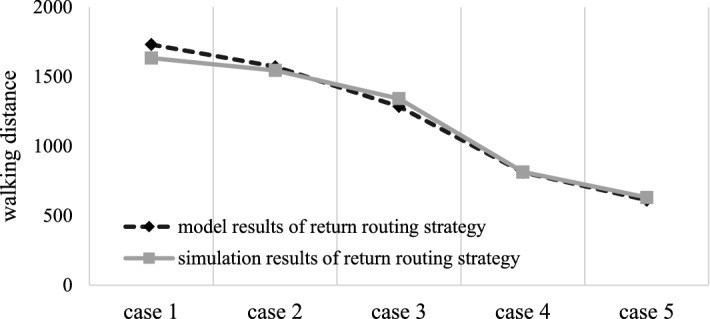
Comparison of the stochastic model results and simulation results for the return routing strategy.

When the routing strategy is return, the model results and simulation results of the above five cases are obtained according to the known conditions, as shown in Table 5 and Fig. 5.

**Table 5:** Model results and simulation results of the return routing strategy.

	Case 1	Case 2	Case 3	Case 4	Case 5
Model results	1731.9	1572.1	1285.6	812.5589	612.1438
Simulation results	1634.3	1545.4	1342.6	814.5706	631.8189
Absolute error	97.6	26.7	− 57	− 2.0117	− 19.6751
Relative error	0.056354	0.016984	− 0.04434	− 0.00248	− 0.03214

The abscissa in Fig. 5 shows five classification cases, and the ordinate represents the distance of the routing strategy. Through the simulation of the model, the following conclusions are obtained. The model results are generally consistent with the simulation results, and the maximum error is approximately 5.64%. It can be considered that the model is consistent with the simulation, which proves that the model is effective. Therefore, it shows that the model can determine that the walking distance of the return routing strategy in the fishbone layout warehouse changes with the different area proportions of classes A, B and C, and that the smaller the area proportion of class A (class B is the second, and class C storage area is the largest), the shorter the walking distance and the greater the operational efficiency of the warehouse.

### Simulation and validation of the S-shape routing strategy

**Figure 6:**
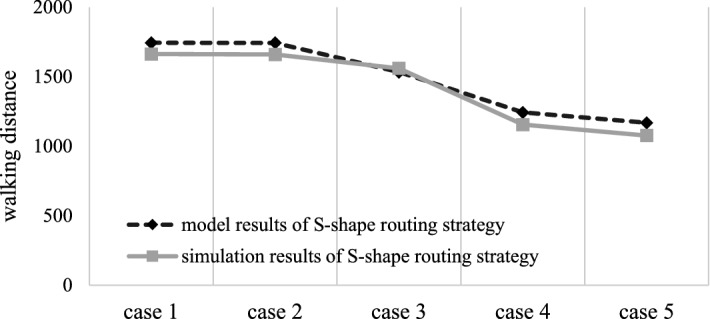
Comparison of the stochastic model results and simulation results for the S-shape routing strategy.

According to the known conditions, when the routing strategy is S-shape, the model results and simulation results of the above five cases are obtained, as shown in Table 6 and Fig. 6.

**Table 6:** Model results and simulation results of the S-shape routing strategy.

	Case 1	Case 2	Case 3	Case 4	Case 5
Model results	1743.8	1743.1	1533	1242.9	1168.5
Simulation results	1663	1659.1	1559.8	1154.8	1076.7
Absolute error	80.8	84	− 26.8	88.1	91.8
Relative error	0.046336	0.04819	− 0.01748	0.070883	0.078562

The abscissa in Fig. 6 shows five classification cases, and the ordinate represents the distance of the routing strategy. Through the simulation of the model, the following conclusions are obtained: the model results are generally consistent with the simulation results, and the maximum error is approximately 7.86%. It can be considered that the model is consistent with the simulation, which proves that the model is effective. Therefore, it shows that the model can determine that the walking distance of the S-shape routing strategy in the fishbone layout warehouse changes with the different area proportions of classes A, B and C, and that the smaller the area proportion of class A (class B is the second, and class C storage area is the largest), the shorter the walking distance and the greater the operational efficiency of the warehouse.

### Comparison of the two routing strategies

**Figure 7:**
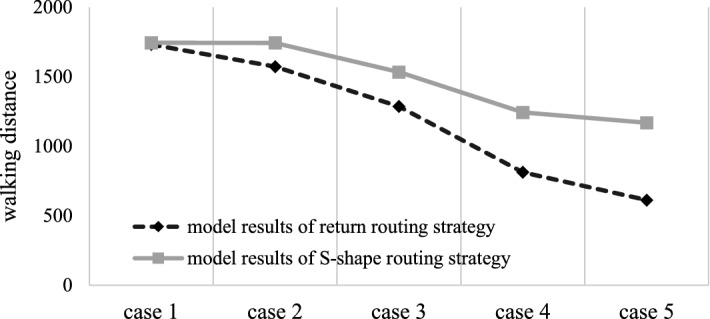
Comparison of model results of the two routing strategies.

**Figure 8:**
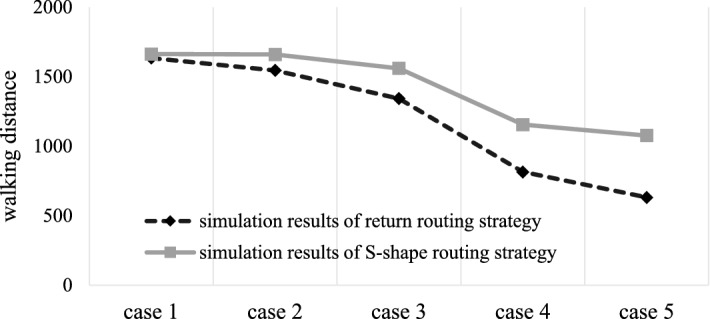
Comparison of simulation results of the two routing strategies.

For the warehouse, two different routing strategies are adopted, the return routing strategy and the S-shape routing strategy. Under the same other constraints, the stochastic model results and simulation results of the two routing strategies are calculated and compared. The results are shown in Tables 7, 8, Figs. 7, and 8.

**Table 7:** Model results of the two routing strategies.

	Case 1	Case 2	Case 3	Case 4	Case 5
Return	1731.9	1572.1	1285.6	812.5589	612.1438
S-shape	1743.8	1743.1	1533	1242.9	1168.5
Absolute error	− 11.9	− 171	− 247.4	− 430.341	− 556.356
Relative error	− 0.00687	− 0.10877	− 0.19244	− 0.52961	− 0.90887

**Table 8:** Simulation results of the two routing strategies.

	Case 1	Case 2	Case 3	Case 4	Case 5
Return	1634.3	1545.4	1342.6	814.5706	631.8189
S-shape	1663	1659.1	1559.8	1154.8	1076.7
Absolute error	− 28.7	− 113.7	− 217.2	− 340.229	− 444.881
Relative error	− 0.01756	− 0.07357	− 0.16178	− 0.41768	− 0.70413

Through the comparative analysis of the model results and simulation results, we find that, when the class-based storage strategy is adopted in the warehouse with the fishbone layout, among the two stochastic models, the maximum difference of order picking walking distance is approximately 90.89% and the minimum difference is approximately 0.69%, while in the simulation results, the maximum difference of order picking walking distance is approximately 70.41% and the minimum difference is approximately 1.76%. Both the model results and the simulation results clearly show that the walking distance of the return routing strategy is shorter. It can be concluded that compared with the S-shape routing strategy, the return routing strategy has better applicability in the fishbone layout warehouse under the class-based storage policy and can improve the operational efficiency of the warehouse to a certain extent.

## Conclusions and future research

By introducing straight lines cutting the storage area of the fishbone layout, this paper divides the fishbone layout warehouse into the set area proportion to meet the requirements of ABC class-based storage. The slope and the intercept are obtained according to equal distances from the I/O point to the boundary of each class area. This lays a foundation for the construction and research of stochastic models. Then, stochastic models of the return and S-shape routing strategies are established for the fishbone layout warehouse, and the effectiveness of the model is verified by simulation using MATLAB. After determining the relevant parameters of the fishbone warehouse layout and orders, the numerical results show that the return routing strategy is better than the S-shape routing strategy.

Research on the routing strategies of fishbone layout warehouses under class-based storage is of great significance both in theory and practice. Based on the analysis of warehouse layout, storage strategy, routing strategies and external order input, the research on walking distance in fishbone layout warehouses can provide theoretical significance for the efficient management and optimal control decision-making of logistics warehouse picking systems, further enriching the theoretical research on logistics warehouse picking, and providing new ideas, methods and theoretical bases for the basic operation and optimization of warehouse picking operations in distribution centers. Proper routing strategies can bring cost advantages to warehouse operations with the fishbone layout, including time and capital. For the current rapidly developing logistics industry, the innovation brought to the operation of warehouses and distribution centers will help improve picking efficiency, shorten the time cost, reduce the capital occupation rate and promote the development of enterprises. For consumers, it can reduce waiting costs and improve service satisfaction.

In the warehouse picking research field, the storage strategy, warehouse layout and routing strategy are all important factors affecting the order picking walking distance, and the effects of the storage strategy, warehouse layout and routing strategy on the order picking efficiency can be comprehensively considered and extended. This paper only reflects one of the combinatorial relationships, which needs further study and research. At the same time, it can be compared with the traditional layout, and then the research content can be verified by the simulation to provide some ideas and directions for future research. In addition, follow-up research on non-traditional layouts under the class-based storage strategy with different criteria could also be considered, for example, not classifying goods solely by turnover rate but also considering the cube-per-order index or the volume of items. And the impact of the number of locations that need to be visited, the number of pickers on the efficiency of picking operations, the storage capacity of goods in the same space, the effectiveness of partition management, and different forms of regional division could be explored in follow-up studies.
